# Characterising the Mechanism of Airway Smooth Muscle β_2_ Adrenoceptor Desensitization by Rhinovirus Infected Bronchial Epithelial Cells

**DOI:** 10.1371/journal.pone.0056058

**Published:** 2013-02-15

**Authors:** David Van Ly, Alen Faiz, Christine Jenkins, Ben Crossett, Judith L. Black, Brent McParland, Janette K. Burgess, Brian G. G. Oliver

**Affiliations:** 1 Woolcock Institute of Medical Research, Sydney, Australia; 2 Respiratory Research Group, Discipline of Pharmacology, The University of Sydney, Sydney, Australia; 3 Department of Respiratory Medicine, Concord General Hospital, Sydney, Australia; 4 School of Molecular Bioscience, The University of Sydney, Sydney, Australia; Cincinnati Children’s Hospital Medical Center, United States of America

## Abstract

Rhinovirus (RV) infections account for approximately two thirds of all virus-induced asthma exacerbations and often result in an impaired response to β_2_ agonist therapy. Using an *in vitro* model of RV infection, we investigated the mechanisms underlying RV-induced β_2_ adrenoceptor desensitization in primary human airway smooth muscle cells (ASMC). RV infection of primary human bronchial epithelial cells (HBEC) for 24 hours produced conditioned medium that caused β_2_ adrenoceptor desensitization on ASMCs without an effect on ASMCs viability. Less than 3 kDa size fractionation together with trypsin digestion of RV-induced conditioned medium did not prevent β_2_ adrenoceptor desensitization, suggesting it could potentially be mediated by a small peptide or lipid. RV infection of BECs, ASMCs and fibroblasts produced prostaglandins, of which PGE_2_, PGF_2α_ and PGI_2_ had the ability to cause β_2_ adrenoceptor desensitization on ASMCs. RV-induced conditioned medium from HBECs depleted of PGE_2_ did not prevent ASMC β_2_ adrenoceptor desensitization; however this medium induced PGE_2_ from ASMCs, suggesting that autocrine prostaglandin production may be responsible. Using inhibitors of cyclooxygenase and prostaglandin receptor antagonists, we found that β_2_ adrenoceptor desensitization was mediated through ASMC derived COX-2 induced prostaglandins. Since ASMC prostaglandin production is unlikely to be caused by RV-induced epithelial derived proteins or lipids we next investigated activation of toll-like receptors (TLR) by viral RNA. The combination of TLR agonists poly I:C and imiquimod induced PGE_2_ and β_2_ adrenoceptor desensitization on ASMC as did the RNA extracted from RV-induced conditioned medium. Viral RNA but not epithelial RNA caused β_2_ adrenoceptor desensitization confirming that viral RNA and not endogenous human RNA was responsible. It was deduced that the mechanism by which β_2_ adrenoceptor desensitization occurs was by pattern recognition receptor activation of COX-2 induced prostaglandins.

## Introduction

Acute exacerbations of asthma are the major cause of morbidity, mortality and health costs related to the disease. Respiratory viral infections trigger approximately 85% of asthma exacerbation in adults and children and the mechanisms by which this occurs remain unclear [1]. Human rhinovirus (RV) belongs to the *Picornaviridae* family of positive single stranded RNA viruses and is implicated in a variety of respiratory disorders ranging from the common cold to the induction of exacerbations of respiratory diseases. Of the respiratory viruses that cause asthma exacerbations, RV accounts for about two thirds of all viral-induced asthma exacerbations [1]. Asthma medications such as corticosteroids and the epinephrine analogues such as selective β_2_ agonists are the most common therapies for asthma management and, during acute exacerbations, including those caused by respiratory viruses, β_2_ agonists are a commonly used rescue medication [2].

Under normal circumstances, airway obstruction in asthma improves in response to inhaled β_2_ agonists, however there have been reports that airway obstruction does not improve with β_2_ agonists during virally induced asthma exacerbations [3], [4]. Reddel and colleagues reported that in asthmatic adults, during a respiratory viral infection their exacerbation was characterized by reduced response to β_2_ agonists despite having good asthma control prior to infection, and a good response to β_2_ agonists prior to achieving good asthma control [3]. Similarly, Rueter et al. reported that asthmatic children responded less effectively to β_2_ agonist therapy in response to a viral-induced exacerbation in which RV was the most frequently identified virus [4]. These reports indicate that the underlying cause of this reduced response to β_2_ agonists during these exacerbations of asthma may be unique to a viral infection. The exact causes of exacerbations of asthma are unknown, however it possible that functional impairment of the β_2_ adrenoceptor (β_2_ AR) may disrupt intrinsic bronchodilation through circulating epinephrine and thus result in airflow limitation characteristic of an exacerbation.

*In vivo*, the epithelial cells form a physiological barrier in the airways and are the principal cell type infected by RV in the lower airways [5] even though there is evidence that underlying submucosal cells can be infected, perhaps as a consequence of viremia or compromised epithelial barrier as occurs in asthma or COPD [6], [7]. Since RV-induced inflammation is thought to contribute to asthma exacerbations, it is likely that the viral-epithelial infection and the subsequent interaction within the epithelial mesenchymal trophic unit are critical in mediating viral-induced exacerbations.

Previous research has suggested that clinical impairment to β_2_ agonist therapy may be due to desensitization of the β_2_ AR on airway smooth muscle cells (ASMCs) [8]. Our group have previously developed an *in vitro* model to show that RV infection of epithelial cells produces a conditioned medium, containing unknown substances, that when applied to ASMCs, causes internalisation of the β_2_ AR, and results in reduced generation of cyclic adenosine monophosphate (cAMP) in response to a β_2_ agonist [8]. Furthermore, the effect observed was not due to the impaired ability to generate cAMP as the adenylate cyclase activator forskolin induced cAMP response was not reduced. This *in vitro* phenomenon may translate to the possible reason why asthmatic patients with RV-induced asthma exacerbations do not respond to β_2_ agonists clinically, however the mechanism by which it occurs, or the identity of the RV-induced epithelial derived substance remains unknown.

Eicosanoids are lipid mediators which incorporate the two large families of prostaglandins and leukotrienes, and their levels are increased in asthma and during clinical RV infections [9], [10]. It has been shown that of the prostaglandin (PG) family, PGE_2_ can cause ASMC relaxation by the induction of cAMP [11]. In doing so, PGE_2_ can cause heterologous desensitization of the β_2_ AR by either activation of common G protein coupled receptor (GPCR) kinases, for example GPCR that share common G_s_ alpha subunits, or alternatively by direct activation of protein kinase A (PKA) [11]–[14]. In contrast, within the leukotriene (LT) family, LTD_4_ causes ASMC contraction by activating cystLT1 receptors which are coupled to G_q_ alpha subunit proteins and activate the protein kinase C (PKC) pathway which ultimately increases intracellular calcium. Surprisingly, LTD_4_ can also cause desensitization of the β_2_ AR [15].

In this study, the identity of the unknown substance/s responsible for or the mechanism that causes β_2_ AR desensitization in ASMCs were investigated using an *in vitro* epithelial-ASM β_2_ AR model using human primary bronchial epithelial cells (HBEC) and human primary ASMCs. In the airways, fibroblasts are structural support cells that lie in close proximity to the epithelium and smooth muscle, and during RV infection could contribute to RV induced β_2_ AR desensitization by their release of mediators such as prostaglandins. For this reason, the ability of airway structural cells including ASMCs, epithelial cells and lung fibroblasts to produce prostaglandins in response to RV infection and the ability of prostaglandins to cause β_2_ AR desensitization in ASMCs were also investigated. We found that RV infection of epithelial cells resulted in an increase in viral RNA which could activate toll-like receptors (TLRs) on the ASMC. This resulted in the generation of COX-2 induced prostaglandins from ASMCs which in turn caused β_2_ AR desensitization. Since RV infection of various airway structural cells also produces prostaglandins, this could further contribute to β_2_ AR desensitization.

## Materials and Methods

### Ethics Statement

The study was approved by the Ethics Review Committee of the Sydney South West Area Health Service, Royal Prince Alfred Hospital and The University of Sydney human research ethics committee. All volunteers provided written informed consent.

### Cell culture: Human Bronchial Epithelial Cells, Airway Smooth Muscle and Fibroblasts

Primary HBEC, ASMC and parenchymal fibroblasts were isolated from macroscopically normal tissue of patients undergoing lung resection for thoracic carcinoma or transplantation for end stage lung disease. All patient details are shown in Table 1 and 2.

**Table 1:** Demographic data of study patients in RV infection of airway structural cells and prostaglandin production.

Patient #	Cell Type	Sex	Age	Disease	Sample
1	Fibroblasts	F	43	Emphysema	Transplant
2	Fibroblasts	F	65	Ca	Resection
3	Fibroblasts	M	68	SCCA	Resection
4	Fibroblasts	F	38	Emphysema	Transplant
5	ASMC	F	65	Ca	Resection
6	ASMC	M	76	SCCA	Resection
7	ASMC	F	59	NSCCA	Resection
8	ASMC	F	66	Ca	Resection
9	ASMC	M	62	Emphysema	Transplant
10	ASMC	F	56	Bronchiectasis	Transplant
11	ASMC	M	47	Emphysema	Transplant
12	ASMC	F	43	Emphysema	Transplant
13	ASMC	M	71	Asthma	Biopsy
14	ASMC	F	49	Asthma	Biopsy
15	ASMC	F	47	Asthma	Biopsy
16	ASMC	M	45	Asthma	Biopsy
17	HBEC	F	54	Emphysema	Biopsy
18	HBEC	M	73	Ca	Resection
19	HBEC	M	64	NSCCA	Resection
20	HBEC	F	72	NSCCA	Resection
21	HBEC	M	63	Emphysema	Transplant

**Table 2:** Demographic data of study patients in the characterisation of the mechanism of RV-induced β_2_ adrenoceptor desensitization.

Patient #	Cell Type	Sex	Age	Disease	Sample
1	HBEC	F	71	Ca	Resection
2	HBEC	M	58	Ca	Resection
3	HBEC	F	60	Emphysema	Transplant
4	HBEC	M	74	Ca	Resection
5	HBEC	F	44	Emphysema	Transplant
6	HBEC	M	66	Ca	Resection
7	HBEC	F	43	Emphysema	Transplant
8	HBEC	M	57	Emphysema	Transplant
9	HBEC	M	58	No disease	Transplant
10	HBEC	F	68	COPD/Ca	Resection
11	HBEC	M	55	Emphysema	Transplant
12	HBEC	F	60	Emphysema	Transplant
13	HBEC	M	54	NSCCA	Resection
14	HBEC	M	58	Emphysema	Transplant
15	HBEC	M	30	Cystic Fibrosis	Transplant
16	HBEC	M	61	Pulmonary fibrosis	Transplant
17	HBEC	M	63	Asthma	Biopsy
18	HBEC	F	51	Emphysema	Transplant
19	HBEC	M	57	NSCCA/COPD	Resection
20	HBEC	F	55	Ca	Resection
21	HBEC	F	59	NSCCA/COPD	Resection
22	ASMC	M	56	α_1_ anti-trypsin deficiency	Transplant
23	ASMC	M	57	α_1_ anti-trypsin deficiency	Transplant
24	ASMC	M	71	Ca	Resection
25	ASMC	M	49	Emphysema	Transplant
26	ASMC	M	33	Asthma	Biopsy
27	ASMC	M	66	SCCA	Resection
28	ASMC	M	38	Asthma	Biopsy
29	ASMC	M	22	No disease	Biopsy
30	ASMC	M	22	Asthma	Biopsy
31	ASMC	M	20	No disease	Biopsy
32	ASMC	M	65	Ca	Resection
33	ASMC	M	21	Asthma	Biopsy
34	ASMC	M	61	Emphysema	Transplant
35	ASMC	M	56	Emphysema	Transplant
36	ASMC	M	22	Asthma	Biopsy
37	ASMC	F	43	Emphysema	Transplant
38	ASMC	F	19	Asthma	Biopsy
39	ASMC	M	65	NSCCA	Resection
40	ASMC	M	41	Emphysema	Transplant
41	ASMC	M	59	Emphysema	Transplant
42	ASMC	M	57	Sarcoidosis	Transplant
43	ASMC	M	66	NSCCA	Resection
44	ASMC	M	70	NSCCA	Resection
45	ASMC	F	45	Emphysema	Transplant
46	ASMC	M	70	Ca	Resection
47	ASMC	M	58	NSCCA/COPD	Resection
48	ASMC	M	59	Ca	Resection
49	ASMC	M	61	Ca	Resection
50	ASMC	M	80	Ca	Resection
51	ASMC	M	75	NSCCA	Resection
52	ASMC	M	72	NSCCA	Resection
53	ASMC	M	64	NSCCA	Resection
54	ASMC	M	43	Pulmonary fibrosis	Transplant
55	ASMC	M	52	Ca	Resection
56	ASMC	M	52	Pulmonary fibrosis	Transplant
57	ASMC	M	59	Emphysema	Transplant
58	ASMC	F	62	Emphysema	Transplant
59	ASMC	M	74	SCCA	Resection
60	ASMC	M	58	Ca	Resection
61	ASMC	F	71	Ca	Resection
62	ASMC	F	29	Pulmonary hypertension	Transplant
63	ASMC	F	44	Emphysema	Transplant
64	ASMC	M	70	Ca	Resection
65	ASMC	F	43	Emphysema	Transplant
66	ASMC	F	56	Emphysema	Transplant
67	ASMC	F	53	Pulmonary fibrosis	Transplant
68	ASMC	M	66	NSCCA	Resection
69	ASMC	M	57	Emphysema	Transplant
70	ASMC	F	59	Ca	Resection
71	ASMC	F	59	Ca	Resection
72	ASMC	M	75	NSCCA/COPD	Resection
73	ASMC	F	41	Ca	Resection
74	ASMC	F	62	Ca	Resection
75	ASMC	M	67	NSCCA	Resection
76	ASMC	F	15	Pulmonary hypertension	Transplant
77	ASMC	M	58	No disease	Transplant
78	ASMC	F	68	NSCCA/COPD	Resection
79	ASMC	M	61	Ca	Resection
80	ASMC	M	55	Emphysema	Transplant
81	ASMC	F	60	Emphysema	Transplant
82	ASMC	M	54	NSCCA	Resection
83	ASMC	F	44	Emphysema	Transplant
84	ASMC	F	51	Pulmonary fibrosis	Transplant
85	ASMC	M	81	No disease	Unknown
86	ASMC	M	71	NSCCA	Resection
87	ASMC	F	50	Emphysema	Transplant
88	ASMC	M	35	Emphysema	Transplant
89	ASMC	M	30	Cystic Fibrosis	Transplant
90	ASMC	M	30	Cystic Fibrosis	Transplant
91	ASMC	F	66	Ca	Resection
92	ASMC	M	53	Emphysema	Transplant
93	ASMC	M	54	Emphysema	Transplant
94	ASMC	M	58	Emphysema	Transplant
95	ASMC	M	64	Emphysema	Transplant
96	ASMC	F	68	Ca	Resection
97	ASMC	M	71	SCCA	Resection
98	ASMC	F	51	Emphysema	Transplant
99	ASMC	M	58	No disease	Biopsy
100	ASMC	M	77	Ca	Resection
101	ASMC	M	60	NSCCA	Resection
102	ASMC	F	58	NSCCA	Resection
103	ASMC	F	67	Ca	Resection
104	ASMC	M	76	Ca	Resection
105	ASMC	F	63	Ca	Resection
106	ASMC	M	26	Bronchiolitis Obliterans	Transplant
107	ASMC	M	66	NSCCA	Resection
108	ASMC	F	62	Emphysema	Transplant

**Key:** **ASMC** = airway smooth muscle cells; **HBEC** = human bronchial epithelial cells; **SCCA = **Small cell carcinoma; **NSCCA** = Non small cell carcinoma; **COPD** = Chronic obstructive pulmonary disease; **Ca** = Cancer.

HBEC and ASMC were obtained from human bronchial airways and fibroblasts from lung parenchyma by methods previously described [16]–[18]. Briefly, segments of bronchus were dissected free from the surrounding parenchyma, cut open and washed in Hanks’ balanced salt solution (HBSS) (Invitrogen, Victoria, Australia). The epithelium was removed and collected into 75 cm^2^ flasks with bronchial epithelial growth medium (BEGM) (Clonetics, California, USA). In doing so this exposes the underlying bands of smooth muscle, which were then gently separated from the underlying connective tissue in small bundles (smooth muscle cell explants). The explants were transferred into 25 cm^2^ flasks and covered with a minimal amount of Dulbecco’s Modified Eagle Medium (DMEM) containing 1% antibiotics: 20 U/L of penicillin, 20 µg/mL of streptomycin, 2.5 µg/mL of amphotericin B, and 10% Fetal Bovine Serum (FBS) (all purchased from Invitrogen). Fibroblasts were isolated from resected lung tissue by seeding 1–2 mm^3^ pieces of parenchymal tissue into DMEM supplemented with 10% FBS and 1% antibiotics in 75 mm^2^ flasks. Both cell types were incubated at 37°C in a humidified atmosphere of 5% CO_2_-95% air.

The medium was replenished every 5 days for the first 10–20 days, and within this time, cell growth occurred. The cells were passaged (split 1∶3) with a solution of trypsin [0.05% wt/vol in HBSS] containing 1 mM EDTA. The cells were maintained in DMEM supplemented with 1% antibiotics in 10% FBS whilst HBEC were maintained in BEGM. All cells were tested for mycoplasma contamination and only mycoplasma free cells were selected and passaged once they reached confluence. ASMCs and fibroblasts at passages 3–7 and HBEC at passages 2–3 were used for experiments.

### RV Propagation and Ultraviolet Inactivation of RV (UVi-RV)

Human RV serotype-16 were propagated in Ohio HeLa cells and purified using a 100,000 kDa molecular weight cut off (MWCO) filter as previously described [19], [20]. RV concentration was determined by virus titration as described previously [21]. In some experiments RV or conditioned medium was UV inactivated in 24 well plates containing 200 ***µ***L of sample/well at a distance of 5 cm from a 30 W UV light source (germicidal lamp G30T8, Sankyo Denki, Japan) for 15 minutes. Successful UV inactivation of RV was established by a virus titration and was used as a non-infectious virus control.

### Rhinovirus Infection of BEC, Fibroblasts and ASMCs and Prostaglandin Production

HBEC were seeded at a concentration of 6.4×10^4^ cells/mL in BEGM, while fibroblasts and ASMCs were seeded at 3.2×10^4^ cells/mL in 10%FBS/DMEM in 6 well plates for 72 hours. The total number of cells after 72 hours was assessed and wells were then either left uninfected (control) or exposed to UV inactivated RV or live RV at a multiplicity of infection (MOI) of 1. The cells were placed on a shaker (100 rpm) for 15 minutes at room temperature before being incubated at 37°C at 5% CO_2_. After 1 hour the cells were washed with HBSS and replaced with fresh BEGM or 10%FBS DMEM before being incubated at 37°C in a humidified atmosphere of 5% CO_2_-95% air. After 24 hours, the supernatant was purged with nitrogen, collected and stored at −80°C for ELISA and the number of remaining viable cells was estimated for each well using trypan blue exclusion and manual cell counting to normalize prostaglandin release to cell number.

### ELISAs

PGE_2_, PGD_2,_ PGF_2α_, PGI_2_ metabolite 6ketoPGF_1α_, CystLT and LTB_4_ ELISAs were purchased from Cayman Chemicals, Michigan, USA. The assays were carried out according to the manufacturer’s instructions. The detection range for each assay was PGE_2_ 7–1000 pg/mL; PGD_2_ 78–5000 pg/mL; PGF_2α_ 3–500 pg/mL; 6ketoPGF_1α_ 1–1000 pg/mL; CystLT 7–1000 pg/mL and LTB_4_ 3–500 pg/mL. Prostaglandin ELISA results were normalised to cell number and expressed as concentration per 1×10^6^cells.

ELISA kits for IL-6 and IL-8 were purchased from R&D Systems (Minneapolis, USA) and BD Biosciences (California, USA) respectively. ELISAs were carried out according to the manufacturers’ instructions. The detection limits of these assays were: 7–1000 pg/mL (IL-6) and 15–2000 pg/mL (IL-8).

### Reagents

Prostaglandins (PG) D_2_, E_2_, F_2α_, the PGI_2_ analogues: MRE-269 (selective) and Beraprost (non-selective) – both of which are being used in clinical trials for the treatment of pulmonary hypertension, the selective COX-2 inhibitor celecoxib (Cayman Chemicals), indomethacin and 3-isobutyl-1-methylxanthine (IBMX) were dissolved in dimethyl sulfoxide (DMSO) (Sigma-Aldrich, Missouri, USA) and stored at −80°C prior to use. Isoprenaline (Sigma-Aldrich) was dissolved in water before use. The protease trypsin was dissolved in Hank’s balanced salt solution (HBSS) (both from Invitrogen) prior to use and to inhibit its digestive activity during experimentation 0.1% bovine serum albumin (BSA) (Invitrogen) was used. The TLR 3 agonist polyinosinic : polycytidylic acid (Poly I:C) (Sigma-Aldrich) and TLR 7/8 agonist imiquimod (InvivoGen, California, USA) were dissolved in DMSO and H_2_O respectively and stored at −20°C. The pro-opiomelanocortin (POMC) protein was purchased ready to use from ABCAM (Cambridge, UK) and the 1.2 kDa “substance X” peptide was synthesized by Auspep (Victoria, Australia) and dissolved in H_2_O. Both protein and peptide were stored at −80°C before use. The prostaglandin receptor antagonists AH6809 (EP_1–3_, DP_1_); CAY10441 (IP); AL8810 (FP); BWA868C (DP_2_); L-161,982 (EP_4_) were purchased from Cayman Chemicals and dissolved in DMSO and stored at −80°C prior to use. In experiments where a single or mix of prostaglandin receptor antagonists or vehicle were used, the final concentrations and formulation for experimentation were chosen according to its use in previous studies to successfully inhibit their specified receptor and they consisted of AH6809 (10^−5^ M) [22]; CAY10441 (10^−6^ M) [23]; AL8810 (10^−5^ M) [24]; BWA868C (10^−5^ M) [25]; L-161,982 (10^−6^ M) [26] or the sum of each corresponding amount of vehicle (DMSO).

### Assessment of Cell Viability and Number

In some experiments, cell viability was assessed by means of measuring mitochondrial activity using the 3-(4,5-dimethylthiazol-2-yl)-2,5-diphenyltetrazolium (MTT) (Sigma) assay as previously described [27]. In other experiments, total cell number was determined by a manual trypan blue exclusion cell count using a haemocytometer and an estimation of the total number of cells was calculated.

### Generation of HBEC Derived Conditioned Medium

Primary HBEC were seeded at a concentration of 5×10^4^ cells/mL in 75 cm^2^ culture flasks with 10 mL of BEGM until >90% confluent. Briefly, RV or UVi-RV (MOI = 2) or BEGM was added to the confluent cell monolayers for 1 hour with orbital shaking at 37°C. The cells were then washed with HBSS and fresh BEGM was added and the cells were incubated at 37°C in a humidified atmosphere of 5% CO_2_ for 24 hours. In experiments with the COX inhibitor indomethacin, infection occurred in the presence of the drug at 1×10^−5^ M in BEGM and replacement BEGM also contained the drug at 1×10^−5^ M. Control, UVi-RV and RV generated conditioned medium was collected 1 day after infection. All conditioned medium (control, UVi-RV and RV) were UV treated by placing 250 µL of conditioned medium into each well of a 12-well tissue culture plate at a distance of 5 cm from a UV lamp for 5 minutes to ensure that all RV was inactivated before being applied undiluted on primary human ASMCs to assess β_2_ AR function.

Primary human ASMCs were incubated with control, UVi-RV and RV conditioned medium for 3 days. In experiments assessing whether prostaglandins, TLR agonists or total extracted RNA from conditioned medium can cause β_2_ AR desensitization, treatments were applied in BEGM or control conditioned medium. In experiments utilizing prostaglandin receptor antagonists to determine the prostaglandin responsible for β_2_ AR desensitization, ASMCs were pretreated with the antagonist 1 hour prior and during the 3 day incubation period with conditioned medium. β_2_ AR function on ASMCs was assessed by the functional cAMP assay and where appropriate ASMC viability was assessed using an MTT assay.

Evidence of epithelial RV infection was assessed by the measurement of RV induced IL-6 by ELISA. The induction of proteins present in RV conditioned medium was assessed using the Quantipro BCA assay kit (Sigma-Aldrich) and 1-D protein gel electrophoresis with silver staining.

### cAMP Assay

To assess β_2_ AR function cells were stimulated with the β agonist isoprenaline (10^−7^ M) for 5 minutes in the presence of the phosphodiesterase inhibitor IBMX (10^−5^ M) in HBSS. The cells were then lysed in a solution of H_2_O with 0.03% (v/v) Tween-20 and 5 mM HEPES buffer by vigorous pipetting. The amount of isoprenaline induced cAMP in the lysate samples was quantified using an Alphascreen cAMP Assay Kit (Perkin Elmer, Massachusetts, USA) according to the manufacturer’s instructions.

### Quantipro BCA Assay Kit

Protein concentration in conditioned medium was determined using the bicinchoninic acid (BCA) assay kit according to the manufacturer’s instruction.

### Protein Gel Electrophoresis

Conditioned medium was prepared as follows for gel electrophoresis: 5 µL of loading buffer (2% sodium dodecyl sulphate (SDS), 7.5% glycerol, 31.25 mM Tris-HCl (pH 6.8), 0.0025% bromophenol blue, 200 mM DTT (all from Sigma-Aldrich) was added to 25 µL of conditioned medium and denatured at 95°C for 5 minutes. Five µL of Precision plus Protein Dual Xtra Molecular Standard ladder (Bio-Rad, New South Wales, Australia) was added in a separate lane of each gel to indicate the size of the visualised bands, while 20 µL of prepared protein samples was added to each separate lane.

Twenty µL of protein samples were loaded onto a 4% polyacrylamide stacking gel (25% (v/v) SDS Tris pH 6.8; 10% (v/v) 37.5∶1 acrylamide (Bio-Rad); 0.1% (v/v) tetramethylethylenediamine (Sigma-Aldrich); 0.1% (v/v) ammonium persulfate (Sigma-Aldrich); in Milli-Q H_2_0) and separated by SDS-polyacrylamide electrophoresis on a 10% polyacrylamide gel (25% (v/v) SDS Tris pH 8.8; 25% (v/v) 37.5∶1 acrylamide; 0.1% (v/v) tetramethylethylenediamine; 0.1% (v/v) ammonium persulfate; in Milli-Q H_2_0) at 150 V for 90 minutes before they were prepared for silver staining. Gels were visualized and analysed using an IS4000MM Kodak imaging system and software (Kodak Scientific Imaging Systems, New York, USA).

### Silver Staining

After protein electrophoresis, the gels were transferred into a 50 mL fixative solution (50% (v/v) methanol, 12% (v/v) glacial acetic acid, 0.05% (v/v) 37% formaldehyde (Sigma-Aldrich), Milli-Q H_2_O) for 30 minutes on an orbital shaker. The fixative solution was then removed and three washes of 50% ethanol in Milli-Q H_2_O were carried out each for 20 minute intervals. The gels were then washed three times with Milli-Q H_2_O and incubated with 20% w/v thiosulfate solution for 1 minute then washed three times again with Milli-Q H_2_O. The gels were incubated in 0.2% w/v silver nitrate in the dark for 20 minutes before being washed twice in Milli-Q H_2_O and then incubated in developer solution (6% (w/v) Na_2_CO_3_, 0.05% (v/v) 37% formaldehyde, 2% (v/v) of thiosulfate solution as described above, in Milli-Q H_2_O) until the bands were clear. The development was stopped by three washes in Milli-Q H_2_O and the bands were captured using the Kodak imaging system.

### Protease Digestion

In order to assess whether RV-induced epithelial derived proteins in the conditioned medium may be responsible for causing β_2_ AR desensitization, large proteins were digested using the protease trypsin and the conditioned medium was reassessed for its ability to induce β_2_ AR desensitization on ASMCs.

Initial experiments involved the optimization of multiple variables including the amount of protease required for digestion, time for sufficient digestion of conditioned medium proteins and the appropriate concentration of inhibitor substance to avoid cytotoxic effects on ASMCs. Protein gel electrophoresis followed by silver staining of digested products validated the digestion process, while MTT assays were used to validate the safety of optimized concentrations of protease and time of digestion on ASMC viability over 3 days. As the protein concentration in conditioned medium varied from batch to batch, control and RV conditioned medium from multiple batches were pooled in these experiments.

Since endogenous proteases are necessary for homeostatic cell function, the use of protease inhibitors to stop the exogenous protease digestion may affect ASMC function and viability. Therefore since trypsin digestion can be safely stopped using saturation with BSA instead of the use of protease inhibitors, trypsin instead of other proteases was selected for enzymatic digestion of the conditioned medium to assess β_2_ AR desensitization.

Briefly, 3–4 batches of control and RV conditioned medium were pooled to a total of 4–6 mL and trypsin was added (500 µg/mL) and the mixture was allowed to incubate in a water bath at 37°C for 24 hours. After the digestion, 0.1% BSA was added to the mixture to saturate and stop the enzymatic digestion. Since the presence of trypsin and BSA may affect the outcome of results, trypsin (>20 kDa) was also inactivated by removal using the Amicon ultra-15 centrifugal filtration unit (Millipore, Massachusetts, USA) with 3 kDa molecular weight cut off (MWCO). The undiluted trypsin digested conditioned medium was applied to ASMCs to assess β_2_ AR desensitization using a functional cAMP assay.

### PGE_2_ Affinity Chromatography Column

PGE_2_ columns filled with 1 mL of PGE_2_ affinity sorbent (mouse anti-PGE_2_ covalently bound to Sepharose 4B) were purchased from Cayman Chemicals. PGE_2_ purification and extraction was carried out according to the manufacturer’s instruction. Briefly, the column was first washed with 0.1 M phosphate buffer solution, and then 4 mL of pooled control or RV conditioned medium was loaded onto the column. The flow-through product free of PGE_2_ (confirmed by PGE_2_ ELISA) was collected for experimentation. The column was then washed twice with 2 mL of distilled water, and a 2 mL solution containing 95% absolute ethanol and 5% distilled water was used to elute the column bound PGE_2_. The eluted solution was completely evaporated by vacuum centrifugation for 2 hours at 37°C and purified PGE_2_ was reconstituted in 1 mL of BEGM for experimentation. Depletion of PGE_2_ was confirmed by ELISA.

### Molecular Weight Fractionation

Amicon ultra-15 centrifugal filtration units of 100, 50, 30, 10, 3 kDa MWCO filters were purchased from Millipore. Initially, 15 mls of control or RV conditioned medium was pooled and loaded into the initial 100 kDa MWCO filter unit and centrifuged at 4000×g for 30 minutes. One mL of the flow-through product was collected for experimentation and the remainder was loaded into the 50 kDa filter unit and centrifuged as before. The sequential filtration, fractionation and sampling were carried out from the 100 kDa to the 3 kDa MWCO filter unit. Flow-through fractions were used to treat ASMCs to assess the relationship between the size fraction and its ability to cause β_2_ AR desensitization.

### Mass Spectrometry

In order to identify whether there was a difference in the peptide profiles between the control and RV conditioned medium that were in the <3 kDa fraction, mass spectrometry (MS) was carried out as previously described by Ly *et al* [28]. In brief, the samples were purified using Ziptips (Millipore) as per the manufacturer’s instructions. The peptides were then analysed by liquid chromatography-mass spectrometry analysis (LCMS). Sample peptides were concentrated and desalted onto a ZORBAX 300SB-C18 trap (5 µm, 5 × 0.3 mm, Agilent Technologies, Victoria, Australia) with 5% (v/v) acetonitrile at 10 µL/min. After a 10 minute wash the pre-column was switched into line with an in-house prepared fritless nano column which had been packed with ReproSil-Pur 120Å C18 (Dr Maisch GmbH, Ammerbuch-Entringen, Germany) and peptides eluted using a acetonitrile gradient over 30 minutes at 300 nL/min. High voltage (2300 V) was applied through a low volume tee (Upchurch Scientific, Washington, USA) at the column inlet and the outlet positioned 1.5 cm from the orifice of a QSTAR Elite hybrid tandem mass spectrometer (Applied Biosystems, California, USA). Positive ions were generated by electrospray and the QSTAR operated in information dependent acquisition mode. A survey scan was acquired (350–1750 m/z, 0.5 s) and the 3 most abundantly multiplied charged ions (counts >30, charge state 2 to 4) sequentially selected by Q1 for MS/MS analysis. Nitrogen was used as the collision gas and optimum collision energy was automatically chosen based on charge state and mass. Tandem mass spectra were accumulated with smart exit enabled (quality = 20 or up to 2 s (65–2000 m/z)).

### Database Searching

Peak lists were generated using Analyst QS 2.0 and were searched using Mascot v2.3 (Matrix Science, Massachusetts, USA) against the LudwigNR 2012 Q1 database. The following criteria were used: precursor and product ion tolerances 0.2 Da; variable modifications of methionine oxidation; trypsin and one allowed missed cleavage specified; and decoy search enabled. Similar searches were performed but with no enzyme specified.

### RNA Purification

Total RNA including micro RNA was purified using the miRNeasy Mini purification kit (Qiagen, Victoria, Australia) as per the manufacturer’s instructions. Total RNA concentration and quality was determined using a spectrophotometer (Nanodrop ND-1000).

### Analysis of Data

All data were checked for normal distribution and when the results were not parametrically distributed, the dataset was log transformed prior to statistical analysis using GraphPad Prism Version 5 (GraphPad Software, California, USA). Results were analysed by paired Student’s t-tests and 1 or 2-way analysis of variance (ANOVA) where appropriate and Bonferroni post test comparison with corresponding controls. In all cases, a *P* value of less than or equal to 0.05 was considered statistically significant.

## Results

### RV-induced Conditioned Medium and β_2_ Adrenoceptor Desensitization

**Figure 1:**
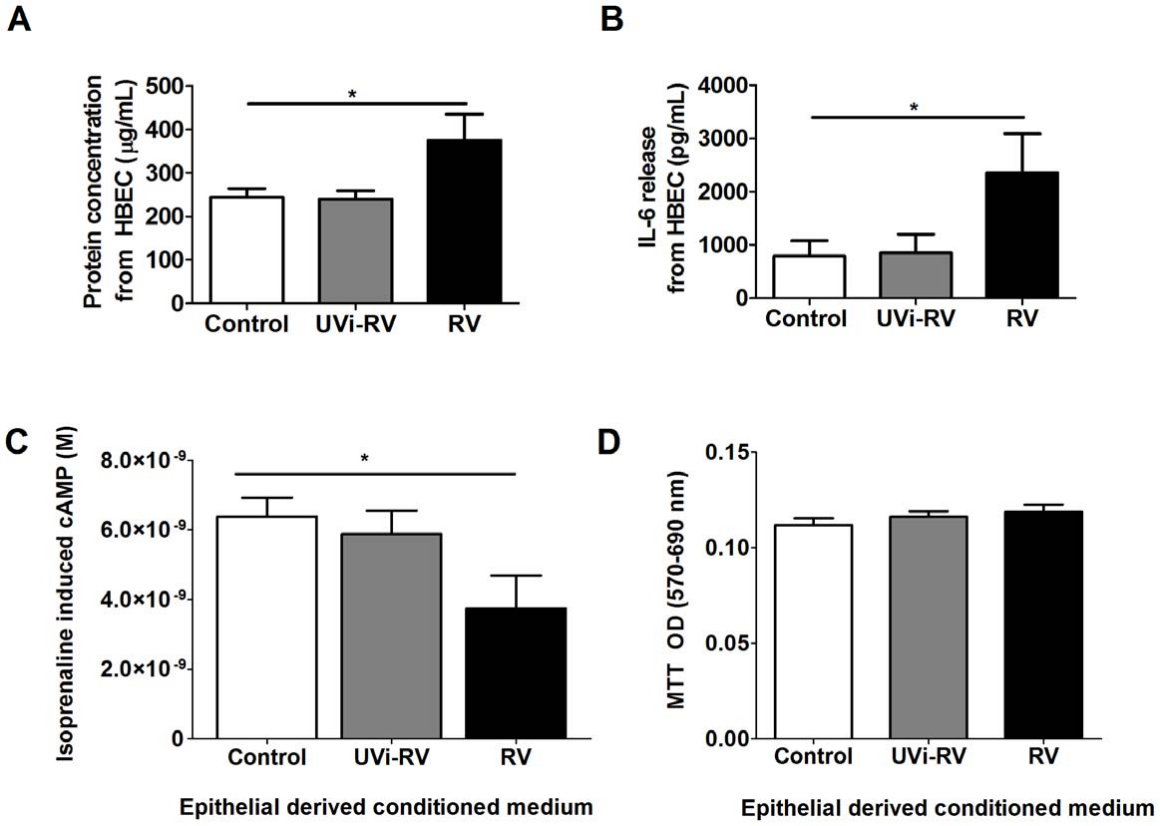
Conditioned medium derived from RV-infected HBEC had increased concentrations of protein and IL-6; and caused a decrease in isoprenaline induced cAMP from ASMCs without an effect on ASMC viability. (A–B) HBEC (n = 4) were uninfected (Control) or exposed to: UV inactivated RV (UVi-RV) or replication competent RV (RV) at an MOI = 2 for 24 hours. The concentration of total protein and IL-6 in the supernatant was measured using a BCA assay and ELISA respectively. (C–D) ASMCs (n = 14) were treated with conditioned medium from HBEC (n = 3) that were uninfected, (Control) or exposed to: UV inactivated RV (UVi-RV) or replication competent RV (RV) at an MOI = 2 for 3 days. Isoprenaline induced cAMP was measured using a cAMP functional assay and ASMC viability was measured using a MTT assay. Data represent mean ± SEM. Statistical differences were examined for using 1-way ANOVA with Bonferroni post test comparison to control treatment *p<0.05.

Conditioned medium was initially characterised and is defined as the supernatant obtained from primary HBEC exposed to no virus (control), UVi-RV or replication competent RV. Since RV infection of HBECs induces IL-6, measurement of IL-6 induction was used as a positive control marker to indicate viral infection [8], [29]. Total protein content and IL-6 concentration in the supernatant from HBEC exposed to UVi RV was not different from the non exposed cells. However supernatant from RV infected HBEC, contained a significant increased total protein content as well as IL-6 concentration compared to unstimulated cells (p<0.05, Figure 1 A-B). Primary ASMCs treated with UVi RV conditioned medium from HBEC did not alter isoprenaline induced cAMP levels compared to control conditioned medium. In contrast RV-induced conditioned medium from HBEC resulted in a decreased isoprenaline induced cAMP from ASMCs compared to treatment with control conditioned medium and therefore was indicative of β_2_ AR desensitization (p<0.05, Figure 1C). RV-induced β_2_ AR desensitization was independent of an effect on cell viability as assessed by MTT (p>0.05, Figure 1D).

### Molecular Weight Fractionation and Trypsin Digestion of Conditioned Medium

**Figure 2:**
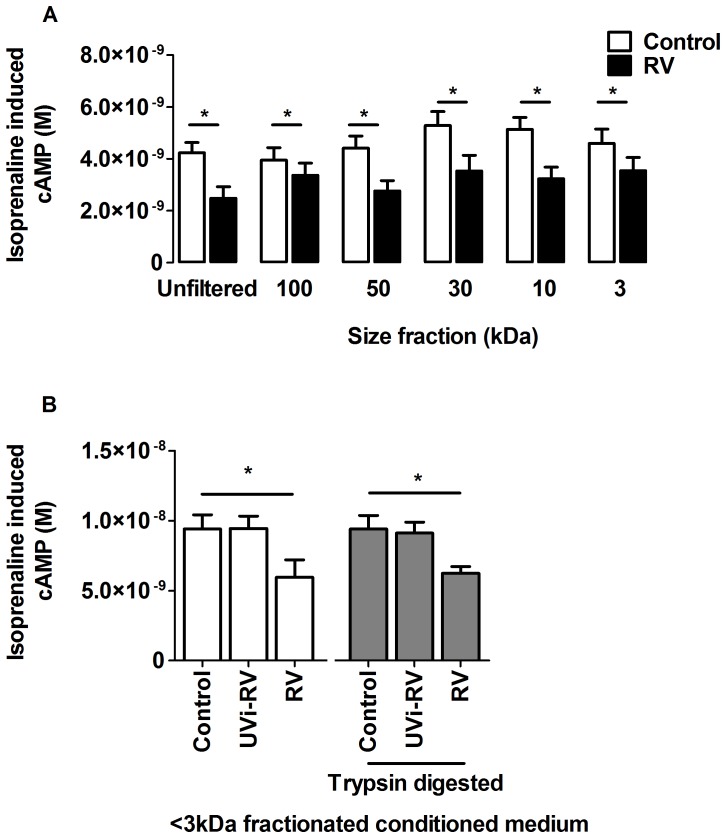
Size fractionation and trypsin digestion of HBEC conditioned medium still resulted in RV-induced β_2_ adrenoceptor desensitization on ASMCs. HBEC (n = 6) were uninfected (Control) or infected with replication competent RV (RV) at an MOI = 2 for 24 hours to generate conditioned medium. The conditioned medium was pooled and serially fractionated through ultrafilters with molecular weight cut offs ranging from 100-3 kDa. Alternatively, conditioned medium from HBEC (n = 3) that was uninfected (Control) or exposed to: UV inactivated RV (UVi-RV) or replication competent RV (RV) at an MOI = 2 for 24 hours was digested in the presence of 500 µg/mL of trypsin for 24 hours at 37°C and trypsin removed using a 3 kDa MWCO ultrafilter. ASMCs (n = 6) were then treated with unfiltered or size fractionated control or RV-induced conditioned medium (A) or 3 kDa fractionated conditioned medium which was undigested or digested with trypsin (B) for 3 days. Isoprenaline induced cAMP was measured using a cAMP functional assay. Data represent mean ± SEM. Statistical differences were detected using multiple paired Student’s t-tests (A) or a 1-way ANOVA with Bonferroni post test (B) comparisons to control conditioned medium *p<0.05.

The RV-induced mediator from HBEC responsible for β_2_ AR desensitization was investigated by size fractionation of the conditioned medium. ASMCs treated with RV-induced conditioned medium that was either not fractioned or was size fractioned through 100, 50, 30, 10 or 3 kDa molecular weight cut off (MWCO) filters, all decreased isoprenaline induced cAMP compared to the control conditioned medium from the corresponding fraction (p<0.05, Figure 2A).

To determine whether the RV-induced mediator was a protein, the conditioned medium was trypsin digested. Complete trypsin digestion was confirmed by absence of detection by ELISA of two different sized proteins IL-6 (26 kDa) and IL-8 (8 kDa) (Figure S1) and further confirmed by the absence of protein bands using protein gel electrophoresis and silver staining (Data not shown).

ASMCs treated with RV-induced conditioned medium that was trypsin digested and fractionated through the 3 kDa filter to remove trypsin still caused decreased isoprenaline induced cAMP compared to their respective controls (p<0.05, Figure 2B). RV-induced conditioned medium that was digested with trypsin and was not fractionated but saturated with BSA to inhibit the activity of trypsin, also caused reduced isoprenaline induced cAMP (Figure S2). These results imply that the mediator responsible for β_2_ AR desensitization was not trypsin digestible and had a molecular weight of <3 kDa.

### RV-induced Release of Prostaglandins from HBECs, ASMCs and Lung Fibroblasts

**Figure 3:**
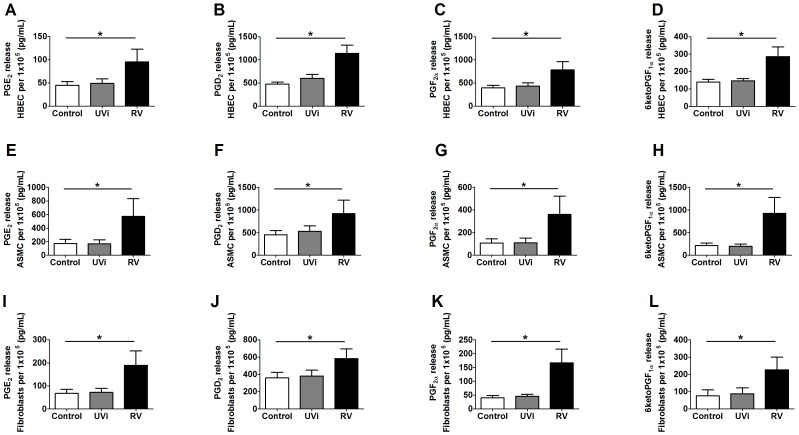
RV infected primary HBECs, ASMCs and lung fibroblasts induced prostaglandins. HBECs (n = 5) (A–D), ASMCs (n = 12) (E–H) and lung fibroblasts (n = 4) (I–L) were uninfected (Control) or exposed to: UV inactivated RV (UVi-RV) or replication competent RV (RV) at an MOI = 1 for 24 hours. Levels of PGE_2_ (A, E, I), PGF_2α_ (B, F, J), PGD_2_ (C, G, K) and the PGI_2_ metabolite 6ketoPGF_1α_ (D, H, L) in the supernatant were measured using ELISA and normalised to total cell number calculated using manual cell counting with trypan blue exclusion after 24 hours. Data represent mean ± SEM. Statistical differences were detected using 1-way ANOVA with Bonferroni post test comparisons to prostaglandin levels in the presence of control conditioned medium *p<0.05.

Since the responsible mediator was <3 kDa and not trypsin digestible, prostaglandin release in response to RV infection was investigated. Primary human HBECs, ASMCs and lung fibroblasts exposed to UVi RV at MOI = 1 did not induced further prostaglandin release compared to basal control levels. RV infection with a MOI = 1 induced the release of PGE_2_, PGF_2α_, PGD_2_ and the PGI_2_ metabolite 6ketoPGF_1α_ from all cell types when compared to basal control levels (p<0.05, Figure 3 A–L).

### Effect of Prostaglandins on β_2_ Adrenoceptor Desensitization

**Figure 4:**
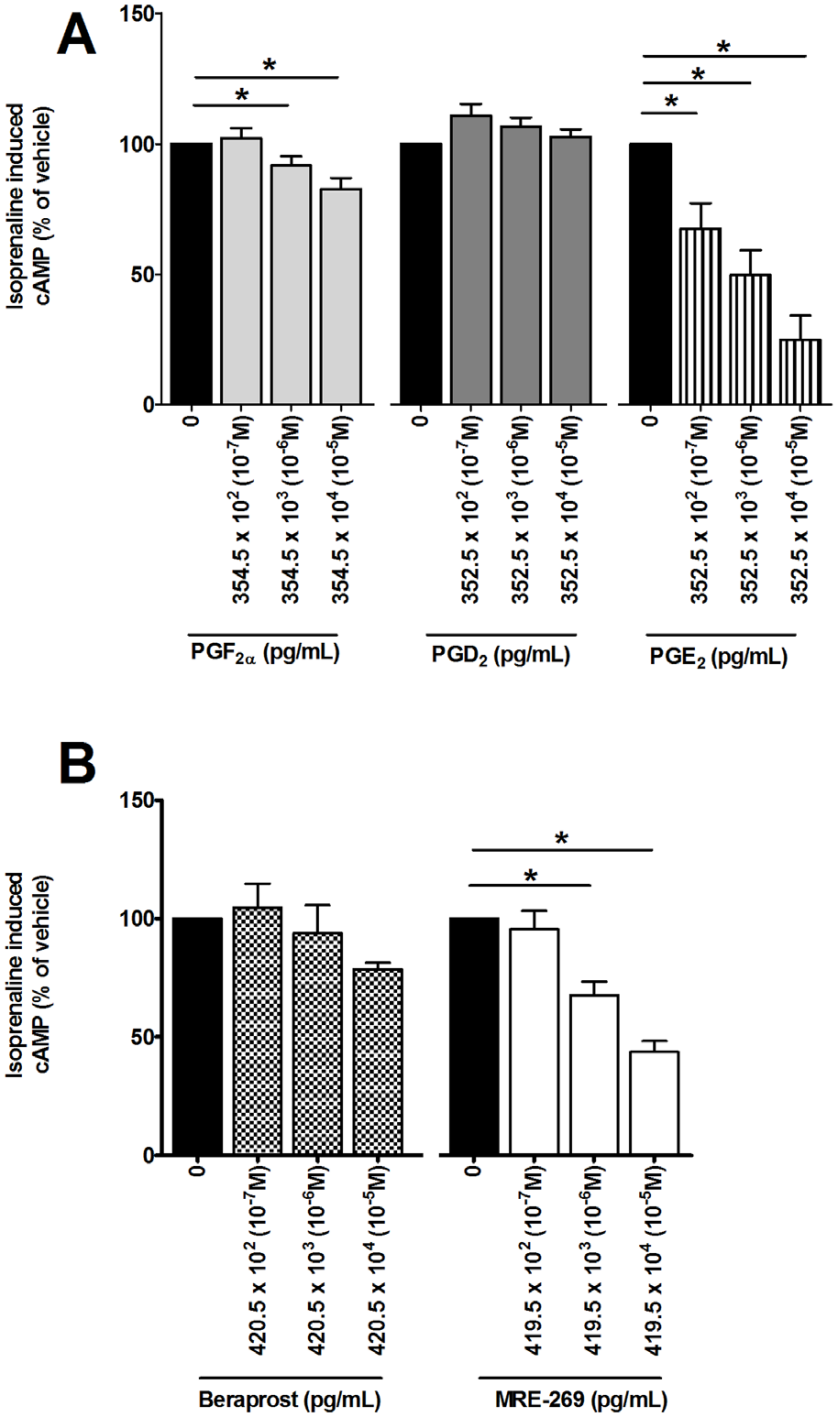
PGF_2α_, PGE_2_, and MRE-269 reduced isoprenaline induced cAMP from ASMCs but not PGD_2_ and Beraprost. ASMCs (n = 8) were treated with vehicle (DMSO) (0 M- *solid black*), PGF_2α_, PGD_2_ and PGE_2_ (10^−7^-10^−5^ M) (*A*) or ASMCs (n = 5) were treated with beraprost and MRE-269 (10^−7^-10^−5^ M) (B) in BEGM for 3 days. Isoprenaline induced cAMP was measured using a cAMP functional assay. Data represent mean ± SEM and are expressed as a percentage of the appropriate vehicle. Statistical differences were detected using 1-way ANOVA with Bonferroni post test comparisons to the respective vehicle *p<0.05.

Since RV induced the release of prostaglandins from primary airway structural cells, their effect on β_2_ adrenoceptor desensitization was investigated. PGE_2_ and PGF_2α_, but not PGD_2_ (10^−7^-10^−5^ M), caused a concentration dependent decrease in isoprenaline induced cAMP compared to the respective vehicle controls (p<0.05, Figure 4A). MRE-269, but not beraprost (10^−7^-10^−5^ M) caused a concentration dependent decrease in isoprenaline-induced cAMP compared to the respective vehicle controls (p<0.05, Figure 4B).

### Effect of Depleting PGE_2_ from Conditioned Medium

**Figure 5:**
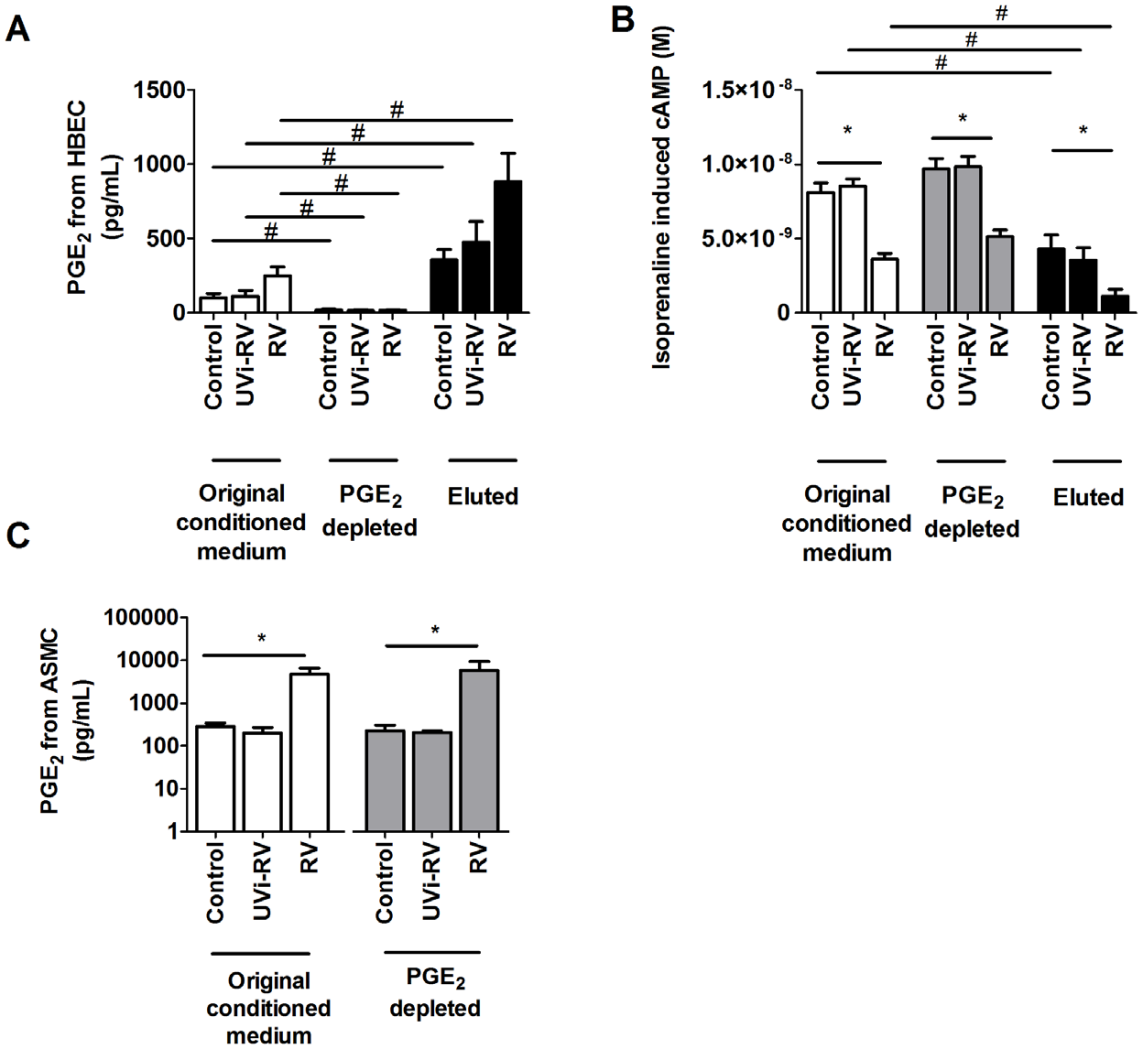
PGE_2_ was successfully depleted from RV-induced conditioned medium from HBEC; but it still caused RV-induced β_2_ adrenoceptor desensitization and further induced PGE_2_ from ASMCs. HBEC (n = 3) were uninfected (Control) or exposed to: UV inactivated RV (UVi-RV) or replication competent RV (RV) at an MOI = 2 for 24 hours to generate conditioned medium. Conditioned medium was depleted of PGE_2_ using affinity chromatography. The original conditioned medium, conditioned medium depleted of PGE_2_ and PGE_2_ eluted product were collected and the levels of PGE_2_ (A) was measured using ELISA. ASMCs (n = 6) were then treated with the original conditioned medium, conditioned medium depleted of PGE_2_ or PGE_2_ eluted product in BEGM for 3 days. Isoprenaline induced cAMP was measured using a cAMP functional assay (B) and the level of PGE_2_ released by ASMCs due to each component was measured using ELISA (C). Data represent mean ± SEM. Statistical differences were detected using a 2-way ANOVA (A&B) and 1-way ANOVA (C) with Bonferroni post test comparisons to the respective control conditioned medium *p<0.05; respective components of the original conditioned medium #p<0.05.

Of the RV-induced prostaglandins, only PGE_2_, PGI_2_ and PGF_2α_ caused β_2_ AR desensitization. With PGI_2_ being unstable and difficult to measure, and the effects of PGF_2α_ on β_2_ AR desensitization being minimal the investigation focused on the role of PGE_2_. Conditioned medium from uninfected, UVi-RV or RV infected HBEC was depleted of PGE_2_ passed through a PGE_2_ immuno affinity column compared with the original conditioned medium (p<0.05, Figure 5A). RV-induced conditioned medium depleted of PGE_2_ still caused decreased isoprenaline induced cAMP from ASMCs (p<0.05, Figure 5B). However the PGE_2_ depleted RV-induced conditioned medium induced the release of PGE_2_ from ASMCs (p<0.05, Figure 5C). The PGE_2_ captured by the affinity column was eluted and was found to be concentrated 4 fold by the extraction process (Figure 5A). Even from the control conditioned medium, the PGE_2_ was sufficient to decrease the isoprenaline induced cAMP from ASMCs and was comparable to that obtained with RV-induced conditioned medium (p<0.05, Figure 5B). Eluted PGE_2_ from RV-induced conditioned medium caused a greater decrease in isoprenaline induced cAMP from ASMCs compared with ASMCs treated with eluted PGE_2_ extracted from control conditioned medium (p<0.05, Figure 5B).

### Effect of COX Inhibitors and Prostaglandin Receptor Antagonists on RV-induced β2 Adrenoceptor Desensitization

The autocrine action of ASMC derived prostaglandins on β_2_ AR desensitization was investigated with pharmacological tools using the non selective COX inhibitor indomethacin, a COX-2 selective inhibitor celecoxib and a combination of PGE_2_, PGD_2_, PGF_2α_ and PGI_2_ receptor antagonists.

**Figure 6:**
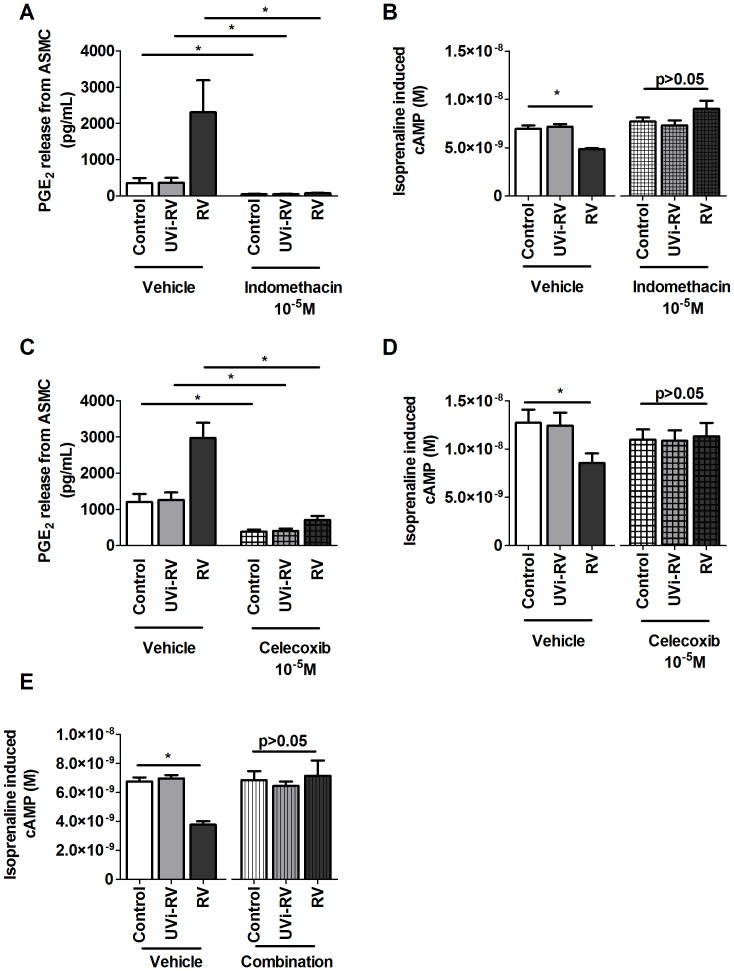
Pharmacological inhibition of prostaglandins prevented the effect of RV-induced β_2_ adrenoceptor desensitization on ASMCs. ASMCs (n = 5) were pretreated for 1 hr with appropriate vehicles or indomethacin (10^−5^ M) (A,B), celecoxib (10^−5^ M) (C,D) or a combination of prostaglandin receptor antagonists (combination composition: AH6809 (10^−5^ M); CAY10441 (10^−6^ M); AL8810 (10^−5^ M); BWA868C (10^−5^ M); L-161,982 (10^−6^ M)) (E) and maintained for a further 3 days in the presence of conditioned medium from HBEC (n = 1) that were uninfected (Control) or exposed to: UV inactivated RV (UVi-RV) or replication competent RV (RV) at an MOI = 2 for 24 hours. PGE_2_ (A, C) was measured using ELISA and isoprenaline induced cAMP (B, D, E) was measured using a cAMP functional assay. Data represent mean ± SEM. Statistical differences were detected using 1-way ANOVA with Bonferroni post test comparisons to control conditioned medium pretreatment in each group *p<0.05.

PGE_2_ release from ASMCs treated with conditioned medium in the presence of indomethacin and celecoxib was significantly attenuated compared to RV-induced conditioned medium in the presence of vehicle (p<0.05, Figure 6 A and C).

ASMCs treated with RV-induced conditioned medium resulted in a decreased isoprenaline induced rise in cAMP in the presence of vehicle (p<0.05), however this effect was absent when ASMCs was treated with indomethacin, celecoxib or a combination of prostaglandin receptor antagonists in the presence of RV-induced conditioned medium (p>0.05 Figure 6 B, D and E).

Since non specific prostaglandin inhibition prevented RV-induced β_2_ AR desensitization, individual receptor antagonists of prostaglandin receptors were used to determine which prostaglandin was responsible for RV-induced β_2_ AR desensitization. However RV-induced conditioned medium still caused β_2_ adrenoceptor desensitization on ASMCs even in the presence of individual PGE_2_, PGI_2_, PGD_2_ or PGF_2α_ receptor antagonists (p<0.05, Figure S3).

### Mass Spectrometry Analysis of Conditioned Medium

In order to investigate whether a small peptide was present in the RV conditioned medium and could be responsible for β_2_ AR desensitization on ASMCs, an overall peptide profile present in the less than 3 kDa fraction of the uninfected, UVi-RV and RV-induced conditioned medium was obtained using mass spectrometry.

A comparative proteomic analysis of uninfected, UVi-RV and RV-induced conditioned medium resulted in the identification of a 1.2 kDa peptide with an 11 amino acid sequence “GDEQPLTENPR” that was present only in the RV-induced conditioned medium. The peptide is a fragment of the pro-opiomelanocortin (POMC) protein, which is a polypeptide that is cleaved to give rise to a super family of neuro-peptides. The 1.2 kDa peptide was synthesized and along with POMC, did not influence ASMC viability, PGE_2_ induction or β_2_ AR desensitization (data not shown).

### RNA Levels in Conditioned Medium and Effect of TLR Activation on β2 Adrenoceptor Desensitization on ASMCs

Toll-like receptors (TLR) are pathogen pattern recognition receptors that are present on ASMCs [30]. Activation of TLRs on ASMCs by small synthetic molecules, pure viral RNA and even fragments of RNA only 5 nucleotides long are immuno-stimulatory [31], [32]. Since activation of the bacterial recognition receptors TLRs 2 & 4 result in the induction of COX-2 induced prostaglandins [33], [34], we investigated whether RV RNA as a result of infection can activate the viral recognition receptors TLR 3, 7 and 8 and also induce COX-2 mediated prostaglandins which may then cause β_2_ AR desensitization on ASMCs.

When conditioned medium generated from HBEC (n = 3) was pooled, the total RNA measured in RV-induced conditioned medium (300.25 ng/µL) was almost 6 times higher than RNA in control conditioned medium (53.91 ng/µL). RV infection produces both ssRNA and dsRNA during replication and whether activation of TLRs 3 and 7/8 using synthetic analogues can result in induction of PGE_2_ and β_2_ AR desensitization on ASMCs were investigated.

**Figure 7:**
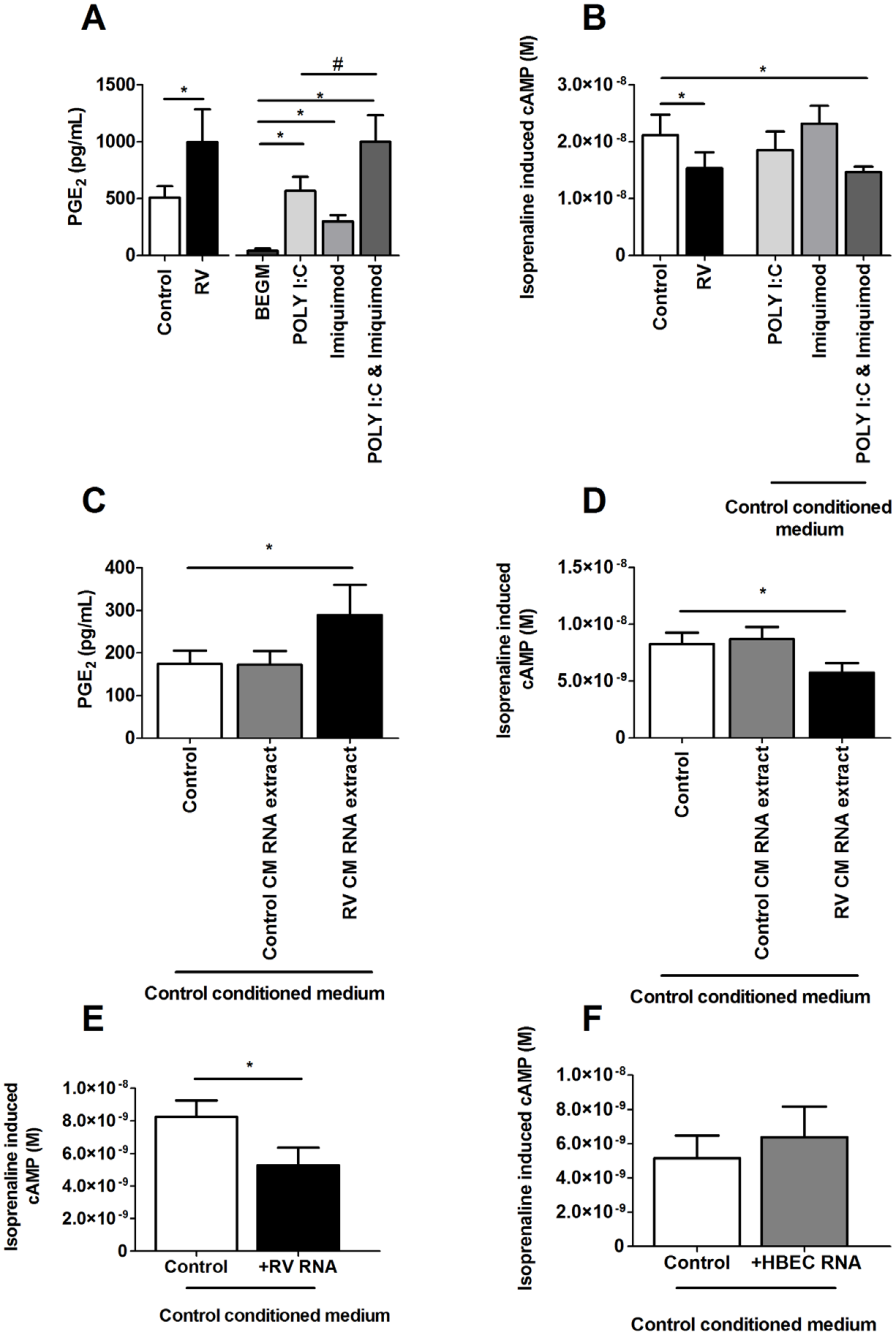
The combination of TLR 3 and 7/8 agonists, and RNA extracted from RV-induced conditioned medium or RV stock caused PGE_2_ induction and β_2_ adrenoceptor desensitization on ASMCs, but not RNA extracted from HBECs. Control and RV-induced conditioned medium was generated from HBEC (n = 2) and pooled. ASMCs (n = 6) were treated with this pooled control or RV-induced conditioned medium; or untreated (BEGM), poly I:C (50 µg/mL), imiquimod (30 µg/mL) and poly I:C & imiquimod (50 µg/mL, 30 µg/mL respectively) in BEGM or in the presence of the control conditioned medium for 3 days (A, B). (Figure C-F) Control and RV-induced conditioned medium was generated from HBEC (n = 3) and pooled. Total RNA was extracted from: control- (53.91 ng/µL), RV-induced conditioned medium (300.25 ng/µL), RV stock (567.35 ng/µL) and cell lysate collected from a sub-confluent 75 cm^2^ flask of HBEC (300 ng/µL) using a miRNeasy Mini purification kit and amount of RNA quantified using a spectrophotometer. ASMCs (n = 6) were treated with pooled control conditioned medium (control), or total extracted RNA collected from those sources in the presence of control conditioned medium for 3 days. PGE_2_ was measured using an ELISA (A, C) and isoprenaline induced cAMP was measured using a cAMP functional assay (B, D-F). Data represent mean ± SEM. Statistical differences were detected using 1-way ANOVA with Bonferroni post test comparisons to respective BEGM, control conditioned medium only (Control) or Poly I:C *p<0.05, #p<0.05.

Treatment with poly I:C (50 µg/mL) or imiquimod (30 µg/mL) induced PGE_2_ from ASMCs compared to BEGM alone (p<0.05) and did not alter isoprenaline induced cAMP (Figure 7 A&B). Treatment with the combination of poly I:C and imiquimod caused an additive induction of PGE_2_ similar to the levels caused by RV-induced conditioned medium from ASMCs and caused a decrease in isoprenaline induced cAMP (p<0.05, Figure 7 A&B).

Treatment with 300.25ng/µL of RNA extracted from RV-induced conditioned medium induced PGE_2_ release and decreased isoprenaline induced cAMP from ASMCs compared to the control treatment without RNA (p<0.05, Figure 7 C&D). These effects did not occur with treatment of RNA extracted from control conditioned medium.

Since the extraction of total RNA from RV induced conditioned medium may include endogenous RNA from HBEC as well as viral RNA, the effect of viral RNA and endogenous RNA on β_2_ AR desensitization was investigated. Treatment with RNA obtained from RV-16 stock in the presence of control conditioned medium decreased isoprenaline induced cAMP from ASMCs compared to the control treatment without RNA (p<0.05, Figure 7E) but treatment with endogenous RNA extracted from HBEC lysate did not alter isoprenaline induced cAMP (p>0.05, Figure 7F) when compared to control conditioned medium without RNA. This suggests that viral RNA in RV induced conditioned medium may be responsible for β_2_ AR desensitization on ASMCs.

## Discussion

Using an *in vitro* model we previously showed that factor(s) released from RV infected epithelial cells cause β_2_AR desensitization on ASMCs [8]. In the present study, we aimed to identify the responsible mediators and pathways which are involved and we confirmed that RV infection of primary HBEC produced conditioned medium that when used to treat ASMCs reduces isoprenaline induced cAMP, thus functionally causing β_2_ AR desensitization. It was deduced that the responsible mediator was less than 3 kDa which meant it could potentially have been a lipid such as a prostaglandin. We showed that RV infected airway cells produce prostaglandins and, since PGE_2_ is known to desensitize the β_2_ AR [11], the effect of other members of the prostaglandin family on the β_2_ AR was examined *in vitro*. It was found that PGF_2α_, PGE_2_ and MRE-269 caused β_2_ AR desensitization and with the use of pharmacological tools it was deduced that β_2_ AR desensitization occurred via autocrine prostaglandins from ASMCs. Mass spectrometry analysis yielded a single peptide present in the less than 3 kDa conditioned medium fraction; however it alone had no influence on β_2_ AR function. Since RNA was detected at greater levels in RV-induced conditioned medium than the control conditioned medium, we investigated RNA as the potential mediator causing β_2_ AR desensitization via the activation of TLR receptors. Using synthetic analogs of double stranded (ds) and single stranded (ss) RNA it was found that their combination caused PGE_2_ induction from ASMCs and β_2_ AR desensitization and similarly, total RNA extracted from RV-induced conditioned medium also caused PGE_2_ induction from ASMCs and β_2_ AR desensitization. Since human endogenous RNA extracted from HBEC did not cause this effect but RNA extracted from RV did, it was deduced that viral RNA produced during RV replication was required to cause β_2_ AR desensitization.

Like all G protein coupled receptors (GPCRs), desensitization of the β_2_ AR occurs physiologically to prevent over activation of the receptor. This can be achieved by the mechanism of homologous desensitization i.e. activation of the receptor by its own defined agonist, for example, activation of the β_2_ AR by a β_2_ agonist causing β_2_ AR desensitization. Alternatively this can also occur by heterologous desensitization i.e. activation of another GPCR resulting in β_2_ AR desensitization because of common downstream cross-regulation of the signal pathways of the G protein subunits [12], [14]. Prostaglandin receptors are all GPCRs, and it is well documented that PGE_2_ can cause heterologous desensitization of the β_2_ AR by means of downstream cross talk of GPCR kinases (GRK) but also by activation of the PKA pathway through the EP_2_ and EP_4_ receptors [12], [13].

Trian et al. first developed this *in vitro* model as used in this study to explore and understand why patients with naturally occurring RV-induced asthma exacerbations do not respond well to β_2_ agonists. We previously deduced that the substance was released only from RV infected HBEC [8]. Findings in the current investigation confirmed the result of Trian et al that RV infection of HBEC indeed produced increased levels of mediators in conditioned medium which may cause β_2_ AR desensitization. In this study our data indicates that the responsible mediator for β_2_ AR desensitization was less than 3 kDa and not affected by trypsin digestion and therefore could potentially be a lipid or a trypsin-resistant peptide.

It has already been reported by Seymour et al that *in vivo* RV infection results in the up regulation of COX-2 enzymes as detected by immunostaining of bronchial mucosal biopsies [9]. However they did not examine which prostaglandin was released or which cell the prostaglandins came from. There are a limited number of studies that have investigated RV-induced eicosanoids from airway structural cells and their impact in the respiratory system. Within this context and of the few investigations in this area, Oliver *et al* reported that RV infection of alveolar macrophages induces the release of PGE_2_ [35]. Kuo *et al* found that RV infection of epithelial cells resulted in the induction of PGE_2_, and RV-induced PGE_2_ could potentially influence airway remodelling via its effect on altering neighbouring cellular behaviour such as cell migration [17]. In the present study we extended the research beyond just PGE_2_, to show that RV infection of primary HBECs, ASMCs and lung fibroblasts can induce the release of various prostaglandin isotypes other than just PGE_2_. The amount of each prostaglandin released in response to RV infection varied amongst the various airway cell types tested, and although this issue is beyond the scope of this study, it could potentially highlight differences in the contribution of each individual prostaglandin as infection progresses from the epithelium to sub-mucosal cells. This raises the possibility of a pool of potential candidates singly or in combination that may be responsible for desensitization of the β_2_ AR.

The investigation showed that RV infection of airway cells produced prostaglandins and PGE_2_ can cause desensitization of the β_2_ AR, which is in keeping with the study by Penn et al. who showed that PGE_2_ can cause desensitization of the β_2_ AR, [11]. Since it has not been previously explored, the role of other prostaglandins was investigated and it was found that PGF_2α_ can also cause desensitization of the β_2_ AR, suggesting it may share common GRKs with the β_2_ AR. Similarly, the PGI_2_ analogue MRE-269 also has the ability to cause β_2_ AR desensitization suggesting this desensitization may involve PKA as well as GRK cross talk. Therefore it is possible that RV infection may result in an increase of prostaglandins in the vicinity and may result in β_2_ AR desensitization on ASMCs. The ability of PGF_2α_ to induce β_2_ AR desensitization was minimal compared to those by MRE-269 and PGE_2_, and since PGI_2_ is extremely unstable and only its inactive metabolite could be measured, it was considered unlikely to be sufficiently stable in the model to cause β_2_ AR desensitization on ASMCs. Since we showed PGE_2_ caused β_2_ AR desensitization most prominently compared to other prostaglandins, it was investigated whether HBEC derived PGE_2_ could be responsible by its depletion from conditioned medium. However, removal of PGE_2_ from conditioned medium did not affect β_2_ AR desensitization on ASMCs. It was then hypothesized that the autocrine action of ASMC derived prostaglandins may instead be responsible for β_2_ AR desensitization on the ASMCs, as PGE_2_ depleted RV-induced conditioned medium further induced approximately 10 fold higher levels of PGE_2_ in comparison to control conditioned medium. This suggested that the unidentified mediator, although not HBEC derived PGE_2_, induces PGE_2_ and potentially other prostaglandins from ASMCs.

To investigate if ASMC derived prostaglandins were responsible for β_2_ AR desensitization, ASMCs was treated with indomethacin and this prevented β_2_ AR desensitization from occurring when ASMCs were exposed to RV-induced HBEC conditioned medium. These findings were similar to the study by Guo et al, who showed that exogenously applied interleukin (IL)-1β induced COX-2 induction of PGE_2_ caused β_2_ AR desensitization and this was also preventable with indomethacin treatment in primary human ASMCs [12]. However IL-1β is a 17 kDa protein and since RV-induced conditioned medium from HBEC does not contain IL-1β and the unknown mediator is smaller than 3 kDa it was excluded as the mediator in this investigation [8], [36], [37]. It was further verified that COX-2 induced autocrine prostaglandins were responsible for β_2_ AR desensitization as treatment with celecoxib also prevented this effect. The hypothesis that autocrine prostaglandins were causing β_2_ AR desensitization was also confirmed when ASMCs treated with a combination of prostaglandin antagonists prevented β_2_ AR desensitization in response to RV-induced HBEC conditioned medium exposure. Therefore this suggests that RV infection of HBEC may be producing unidentified mediators which cause increased activity of COX-2 and production of prostaglandins from ASMCs which act autocrinely to cause β_2_ AR desensitization.

An attempt was made to determine which prostaglandin isotype was responsible for β_2_ AR desensitization by pre-treating ASMCs with different prostaglandin receptor antagonists only to find that none of the antagonists alone prevented β_2_ AR desensitization. The receptor antagonists available are designed to be selective for individual prostaglandin receptors however, in some circumstances; the antagonists are not selective enough and lack the ability to differentiate between the very similar receptors of PGE_2_ and PGD_2_. In addition, prostaglandins are also very pleotropic and can cross activate each other’s receptors [38], [39]. In future, with more defined and specific antagonists available or by genetically knocking down specific prostaglandin receptors, it may be possible to delete individual autocrine prostaglandins and to identify the combination of prostaglandin isotypes involved in RV-induced β_2_ AR desensitization.

Using mass spectrometry it was found that a 1.2 kDa peptide was present in RV-induced conditioned medium, but not in the control conditioned medium. The 1.2 kDa peptide is a fragment from the POMC protein which is a precursor for various hormones and ultradian rhythm neuro-peptides [40]. However treatment of ASMCs with the 1.2 kDa peptide or the polypeptide POMC did not cause β_2_ AR desensitization on ASMCs, suggesting they are not the responsible mediators or that a combination of mediators are required.

Since RV-induced conditioned medium cause β_2_ AR desensitization through an immuno-stimulatory process such as via the process of autocrine COX-2 induction, and the unidentified mediator was small, it was hypothesized that the responsible mediator might be viral RNA or viral RNA fragments.

Results of the study showed that the use of the synthetic TLR 7/8 agonist imiquimod and the TLR 3 agonist poly I:C alone slightly increased PGE_2_ release from ASMCs but did not alter isoprenaline induced cAMP. This result is in agreement with that of Cooper et al, who also showed that poly I:C promotes inflammatory mediator release [41]. However in order to mimic the presence of both viral ssRNA and dsRNA fragments which would occur during RV replication, the combination of both synthetic agonists of the TLRs 3 and 7/8 was used and it was found that it caused β_2_ AR desensitization as well as an additive induction of PGE_2_ from ASMCs equivalent to those levels produced by RV-induced conditioned medium. This was further explored by showing that RNA extracted from an equal volume of RV-induced HBEC conditioned medium was higher than the control conditioned medium, and that RNA collected from RV-induced conditioned medium caused ASMC β_2_ AR desensitization. Total RNA extracted from RV-induced conditioned medium could include both endogenous human RNA as well as RV RNA and it is difficult to distinguish these small nucleotide fragments. It was confirmed that equal concentrations of RNA extracted from pure RV-16 caused PGE_2_ induction and β_2_ AR desensitization on ASMCs but this was not the case for endogenous human RNA extracted from HBEC lysate, which suggests that foreign viral RNA is required.

RV is a positive sense ssRNA virus and during replication, dsRNA are formed in order to generate more copies of the ssRNA genome to be packed into new virions. However, like many RNA viruses, RV replication is susceptible to RNA polymerase error with a rate of 10^−3^ and 10^−4^ errors/nucleotide/cycle of replication [42]. This could result in incorrect or incomplete formation of new ssRNA strands which may not be packed into new virion capsules and may accumulate intracellularly. Continuing RV replication would eventually result in the accumulation of large amounts of both incomplete and complete viral ss/dsRNA inside the cell. This viral RNA could escape the cell via transporting vesicles, cellular pores or upon cell lysis which could expose foreign RV RNA as well as new infectious RV progeny to neighbouring cells. In the case of the ASMC, it is possible that RV RNA could potentially trigger TLRs to activate COX-2 induced prostaglandins and therefore result in β_2_ AR desensitization. Whilst UVi-RV contains RV RNA, the virions cannot replicate and therefore there is no increase in viral RNA. Whether β_2_ AR desensitization is restricted to asthma, or not is not known. It has been reported that greater RV replication occurs in epithelial cell derived from people with asthma [43] so there is a possibility that there would be greater RV RNA. Alternatively, it is possible that in people without asthma or airway hyper-reactivity, RV-induced β_2_ AR desensitization can occur during colds and remains clinically unnoticed. It is uncertain whether full length RV RNA or RNA fragments activate ASMCs to cause β_2_ AR desensitization, however both are possibilities, as it has been shown that nucleotide fragments as small as 5 nucleotides long can activate TLR 7/8 [32].

This *in vitro* model attempted to simulate the events of an *in vivo* RV infection of the lung, which occurs principally in the airway epithelium and may result in ASMC β_2_ AR desensitization. However there are limitations to the current model that need to be acknowledged. Primarily, the *in vitro* model involved only 2 cell types, whilst physiologically there could be more cell types involved during infection. For example inflammatory cells and or perhaps other structural cells such as fibroblasts could play a role. For this reason it is possible that other cells may be as influential in causing β_2_ AR desensitization on ASMCs as epithelial cells. Furthermore, when other cells are considered, the system can become much more complex, and protein mediators such as IL-1β which can also cause β_2_ AR desensitization [12], [44] may also be involved. In addition, as it was shown that each size fraction caused β_2_ AR desensitization, it does not necessarily mean that it is only one small mediator that was responsible. It is possible that there is a combination of multiple sized mediators trapped in each fraction which could activate GPCRs and cause β_2_ AR desensitization.

Although it was deduced in the current model that β_2_ AR desensitization was due to RV RNA activating TLRs, in our investigation RNA was extracted using a trizol methodology and for this reason small RNA mediators such as RV-induced HBEC derived micro (mi) RNA which may be able to activate TLRs [32] and/or have their own undefined effect on β_2_ AR modulation could also be responsible and requires further investigation. It is theoretically possible to degrade the RNA in RV-induced conditioned medium using exogenous ribonucleases and assess for β_2_ AR desensitization. The limitation to this is that RNA that has been degraded to as small as 5 nucleotides long may still be or become immuno-stimulatory [32]. In a multicellular environment, production of leukotrienes is also highly likely and can also cause β_2_ AR desensitization, as it has been shown previously with LTD_4_ [15]. In addition, cytoplasmic helicase proteins such as retinoic-acid-inducible protein I (RIG-I) and melanoma-differentiation-associated gene 5 (MDA5) have been implicated in viral dsRNA recognition [45]. During RV infection of epithelial cells TLR activation is responsible for co-ordinating the up regulation of RIG-I and MDA-5 which further induces antiviral responses [46]. Therefore, there is a possibility that activation of TLRs but also RIG-I and MDA-5 by RV RNA could potentially contribute in a co-ordinated manner to induce β_2_ AR desensitization and should be investigated in future studies.

The second limitation of the *in vitro* model is the use of primary human ASMCs and BEC which were obtained from multiple disease tissue origins. The use of primary human cells is a better representation of human cellular behaviour than some transformed cell lines because they reflect more accurately the true nature of biological heterogeneity of cellular responses, however since the current model utilized two cell types which were not diseased matched, and in some cases, HBEC derived conditioned medium was pooled from multiple diseases, it might be possible that asthma specific effects were missed.

Results from the present study identify the potential mediator responsible for RV-induced β_2_ AR desensitization as viral RNA. During RV replication, viral RNA increases and activates TLRs and as a result induces COX-2 mediated prostaglandin production which causes β_2_ AR dysfunction. Interestingly, the findings in this study also raise the possibility that β_2_ AR desensitization may not be entirely unique to RV infection but could also be caused by other respiratory viruses that actively infect and replicate. By extrapolating the current findings to the clinical situation, the potential use of COX-2 inhibitors to restore β_2_ agonist efficacy in asthmatic patients during viral infection is possible and could be investigated clinically.

## Supporting Information

Figure S1 **Trypsin digestion of conditioned medium removes IL-6 and IL-8.** Conditioned medium pooled from HBEC (n = 3) that were uninfected (Control) or treated with: UV inactivated RV (UVi-RV) or replication competent RV (RV) at an MOI = 2 for 24 hours was digested in the presence of 500 µg/mL of trypsin for 24 hours at 37°C and the reaction was stopped with 0.1% BSA. IL-6 and IL-8 were measured using ELISA with n = 1 experimental repeat.
(TIF)

Figure S2 **Trypsin digestion of conditioned medium still causes ASMC β_2_ adrenoceptor desensitization.** Conditioned medium pooled from HBEC (n = 3) that were uninfected (Control) or exposed to: UV inactivated RV (UVi-RV) or replication competent RV (RV) at an MOI = 2 for 24 hours was digested in the presence of 500 µg/mL of trypsin for 24 hours at 37°C and the reaction was stopped with 0.1% BSA. ASMCs (n = 6) were treated with trypsin digested conditioned medium for 3 days. Isoprenaline induced cAMP was measured using a cAMP functional assay. Data represent mean ± SEM. Statistical differences were detected using a 1-way ANOVA with Bonferroni post test comparisons to control conditioned medium *p<0.05.
(TIF)

Figure S3 **Individual prostaglandin antagonists do not prevent β_2_ adrenoceptor desensitization.** ASMCs (n = 6) were pretreated for 1 hr with vehicle (0.1% DMSO), CAY10441 (10^−6^ M) (A), AH6809 (10^−5^ M) (B), AL8810 (10^−5^ M) (C), BWA868C (10^−5^ M) (D) or L-161,982 (10^−6^ M) (E) and maintained for a further 3 days in the presence of conditioned medium from HBEC (n = 2) that were uninfected (Control) or infected with replication competent RV (RV) at an MOI = 2 for 24 hours. Isoprenaline induced cAMP was measured using a cAMP functional assay. Data represent mean ± SEM. Statistical differences were detected using 1-way ANOVA with Bonferroni post test comparisons to the control conditioned medium pretreatment in each group *p<0.05.
(TIF)
