# Supplementary figures and images for: Characterising the Mechanism of Airway Smooth Muscle β2 Adrenoceptor Desensitization by Rhinovirus Infected Bronchial Epithelial Cells
Source: PLoS One. 2013 Feb 15;8(2):e56058. doi: 10.1371/journal.pone.0056058 (PMC3574065; doi:10.1371/journal.pone.0056058)

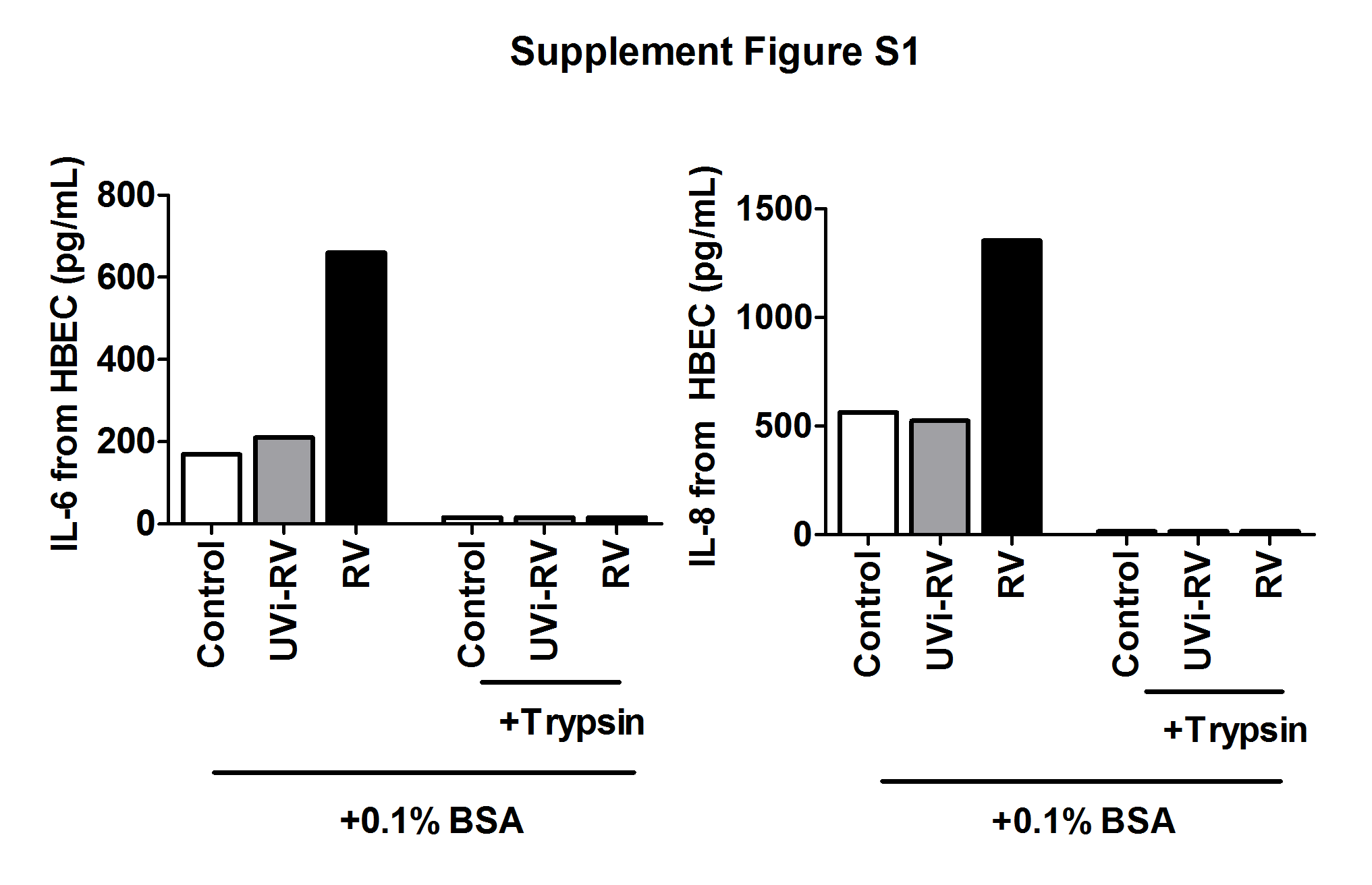

Supplement: Figure S1 — Trypsin digestion of conditioned medium removes IL-6 and IL-8. Conditioned medium pooled from HBEC (n = 3) that were uninfected (Control) or treated with: UV inactivated RV (UVi-RV) or replication competent RV (RV) at an MOI = 2 for 24 hours was digested in the presence of 500 µg/mL of trypsin for 24 hours at 37°C and the reaction was stopped with 0.1% BSA. IL-6 and IL-8 were measured using ELISA with n = 1 experimental repeat. (TIF) [file pone.0056058.s001.tif]

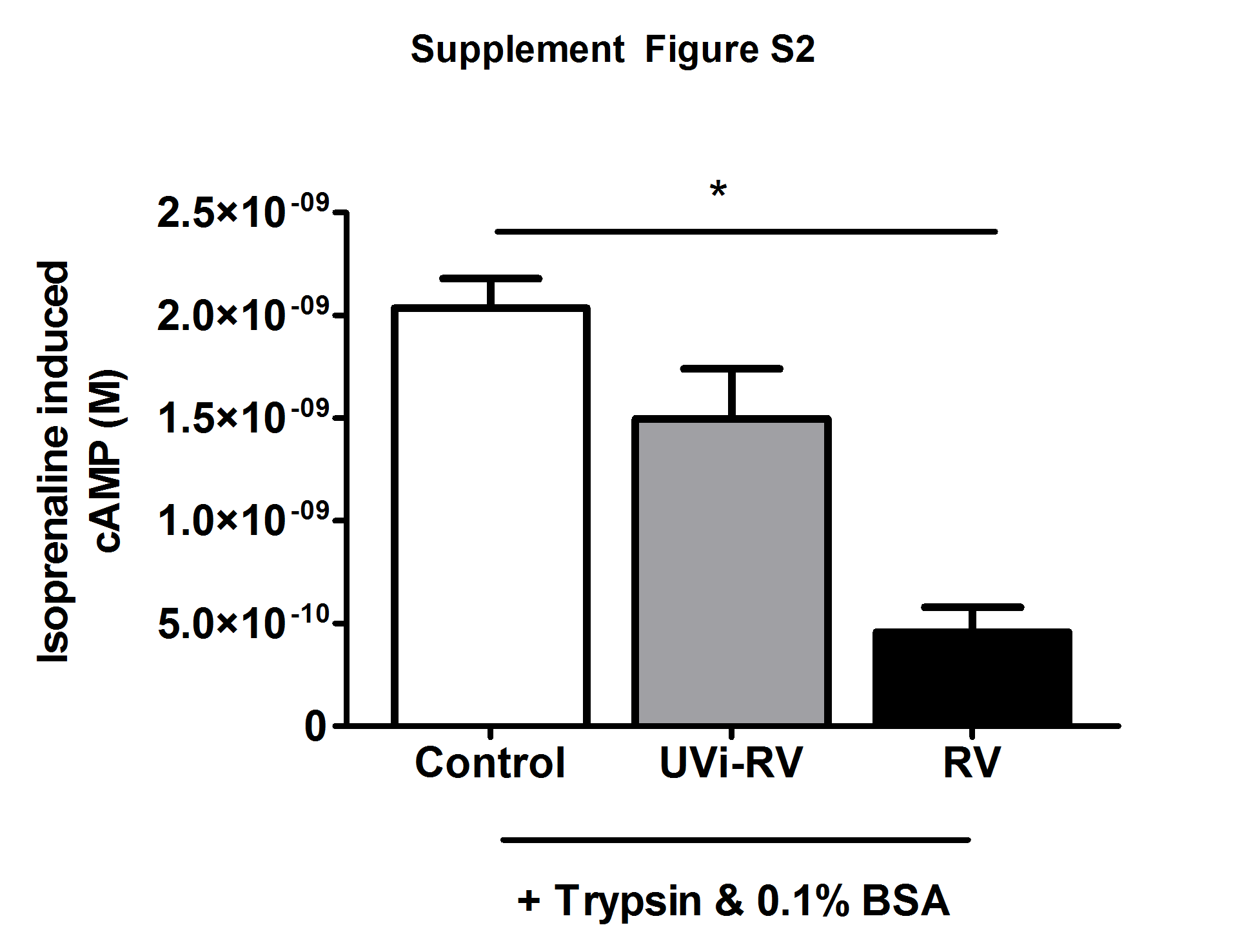

Supplement: Figure S2 — Trypsin digestion of conditioned medium still causes ASMC β2 adrenoceptor desensitization. Conditioned medium pooled from HBEC (n = 3) that were uninfected (Control) or exposed to: UV inactivated RV (UVi-RV) or replication competent RV (RV) at an MOI = 2 for 24 hours was digested in the presence of 500 µg/mL of trypsin for 24 hours at 37°C and the reaction was stopped with 0.1% BSA. ASMCs (n = 6) were treated with trypsin digested conditioned medium for 3 days. Isoprenaline induced cAMP was measured using a cAMP functional assay. Data represent mean ± SEM. Statistical differences were detected using a 1-way ANOVA with Bonferroni post test comparisons to control conditioned medium *p<0.05. (TIF) [file pone.0056058.s002.tif]

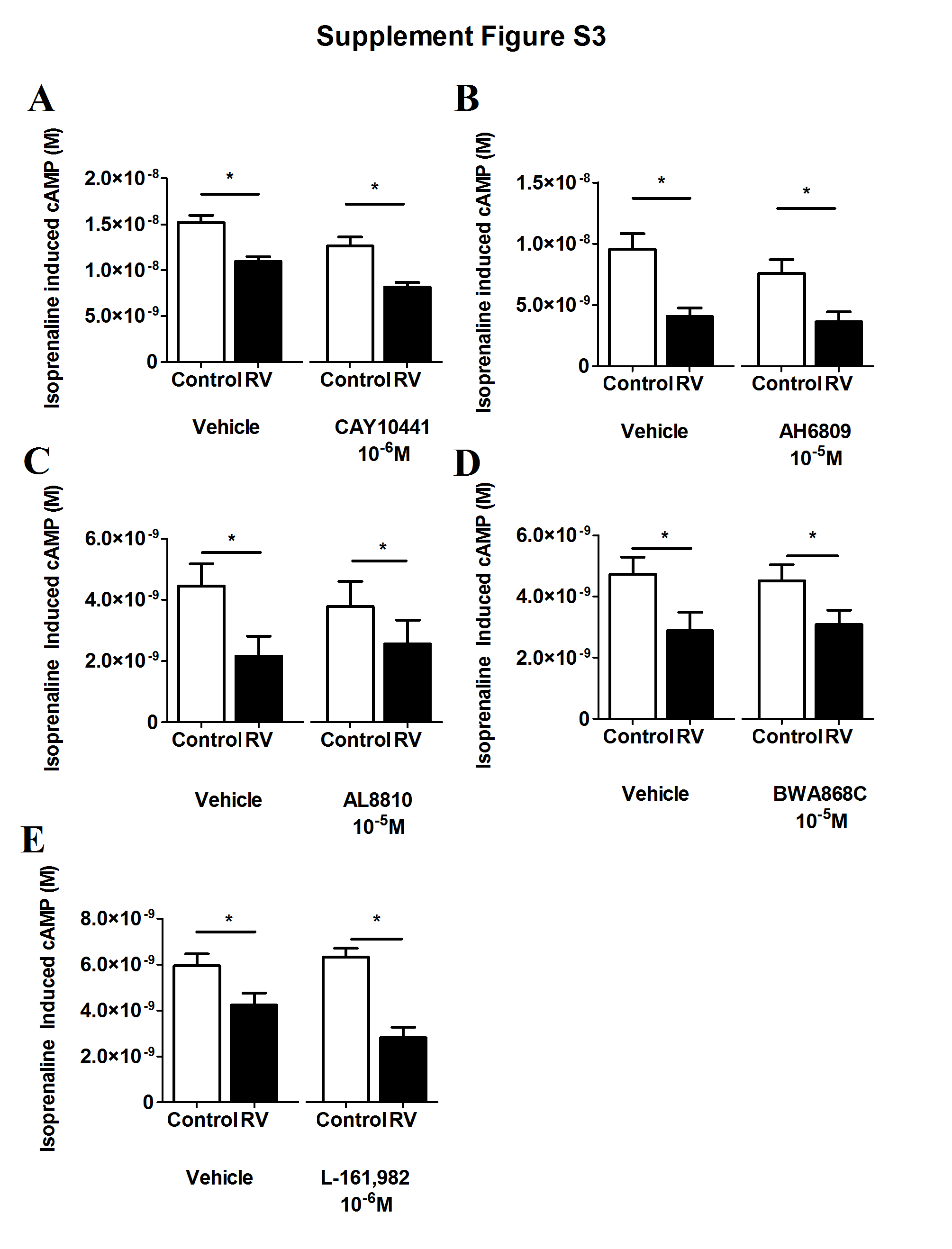

Supplement: Figure S3 — Individual prostaglandin antagonists do not prevent β2 adrenoceptor desensitization. ASMCs (n = 6) were pretreated for 1 hr with vehicle (0.1% DMSO), CAY10441 (10−6 M) (A), AH6809 (10−5 M) (B), AL8810 (10−5 M) (C), BWA868C (10−5 M) (D) or L-161,982 (10−6 M) (E) and maintained for a further 3 days in the presence of conditioned medium from HBEC (n = 2) that were uninfected (Control) or infected with replication competent RV (RV) at an MOI = 2 for 24 hours. Isoprenaline induced cAMP was measured using a cAMP functional assay. Data represent mean ± SEM. Statistical differences were detected using 1-way ANOVA with Bonferroni post test comparisons to the control conditioned medium pretreatment in each group *p<0.05. (TIF) [file pone.0056058.s003.tif]
